# Supplementary material for: Inhibiting stearoyl-CoA desaturase suppresses bone metastatic prostate cancer by modulating cellular stress, mTOR signaling, and DNA damage response
Source: FEBS Lett. Author manuscript; Available in PMC 2026 Mar 8. (PMC12967245; doi:10.1002/1873-3468.70290)
Supplement: Supplementary Figures [file NIHMS2146576-supplement-Supplementary_Figures.pdf]

SCD Expression in Patient Samples

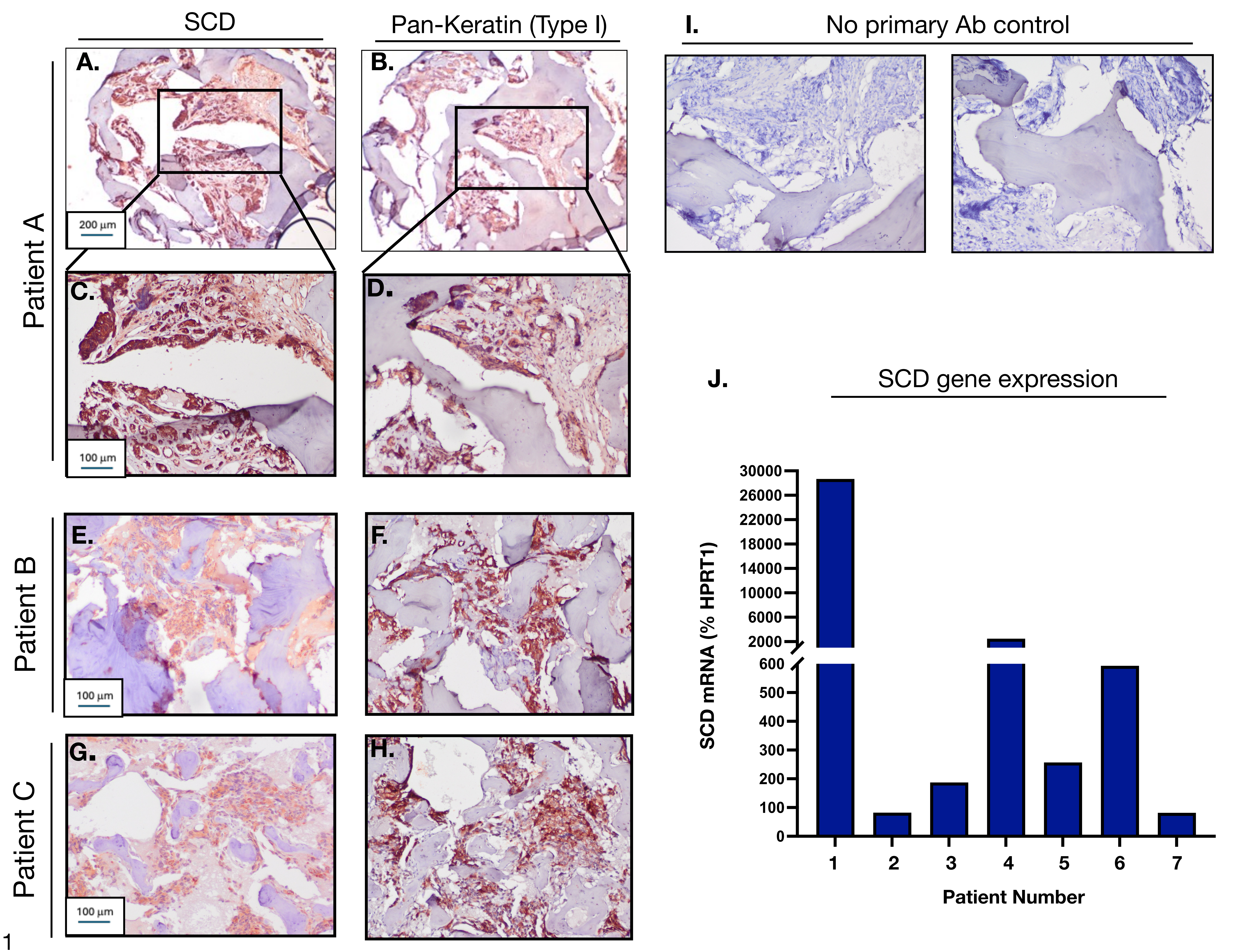

Supplementary Fig. 1

siRNA-mediated SCD knockdown

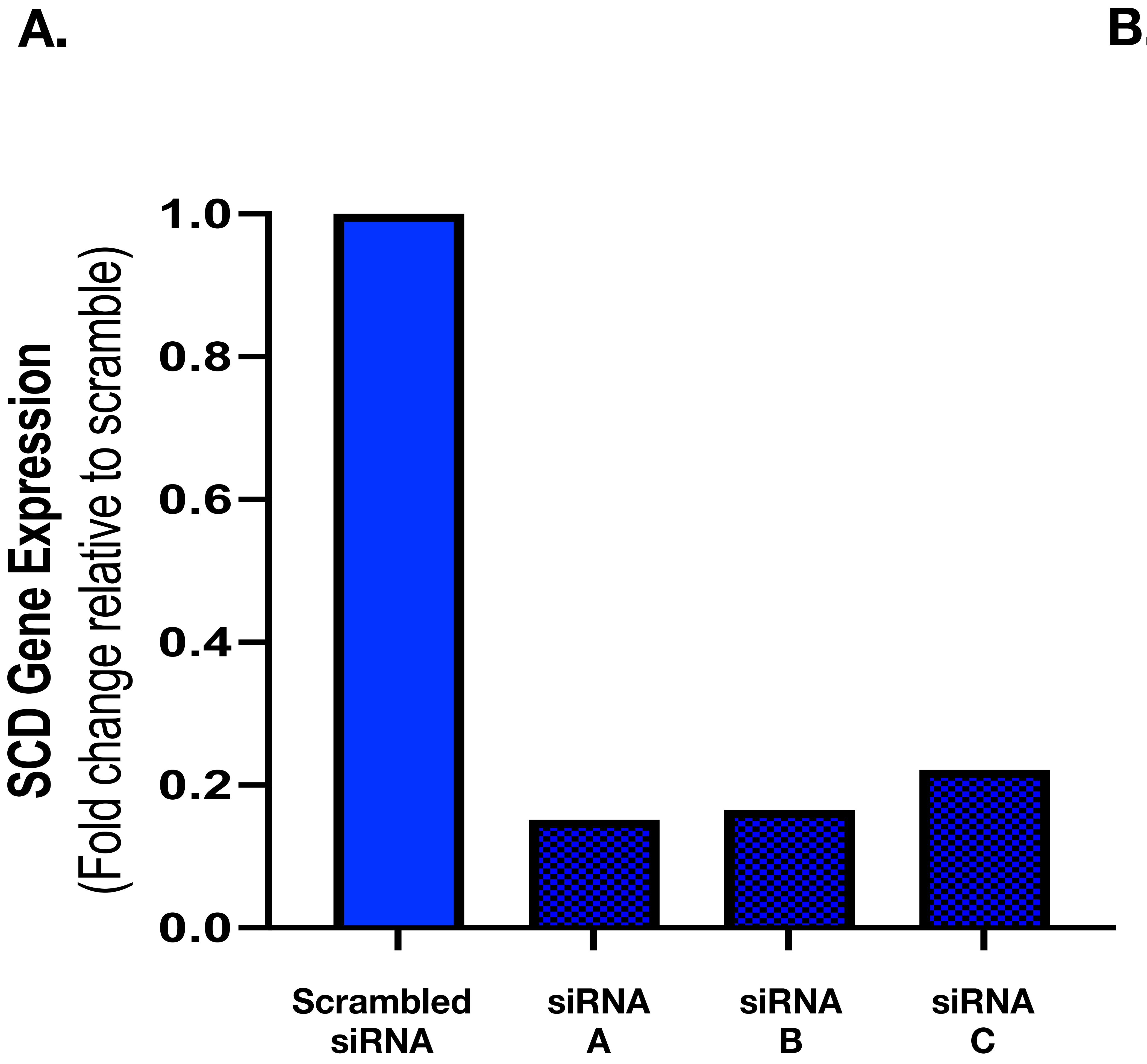

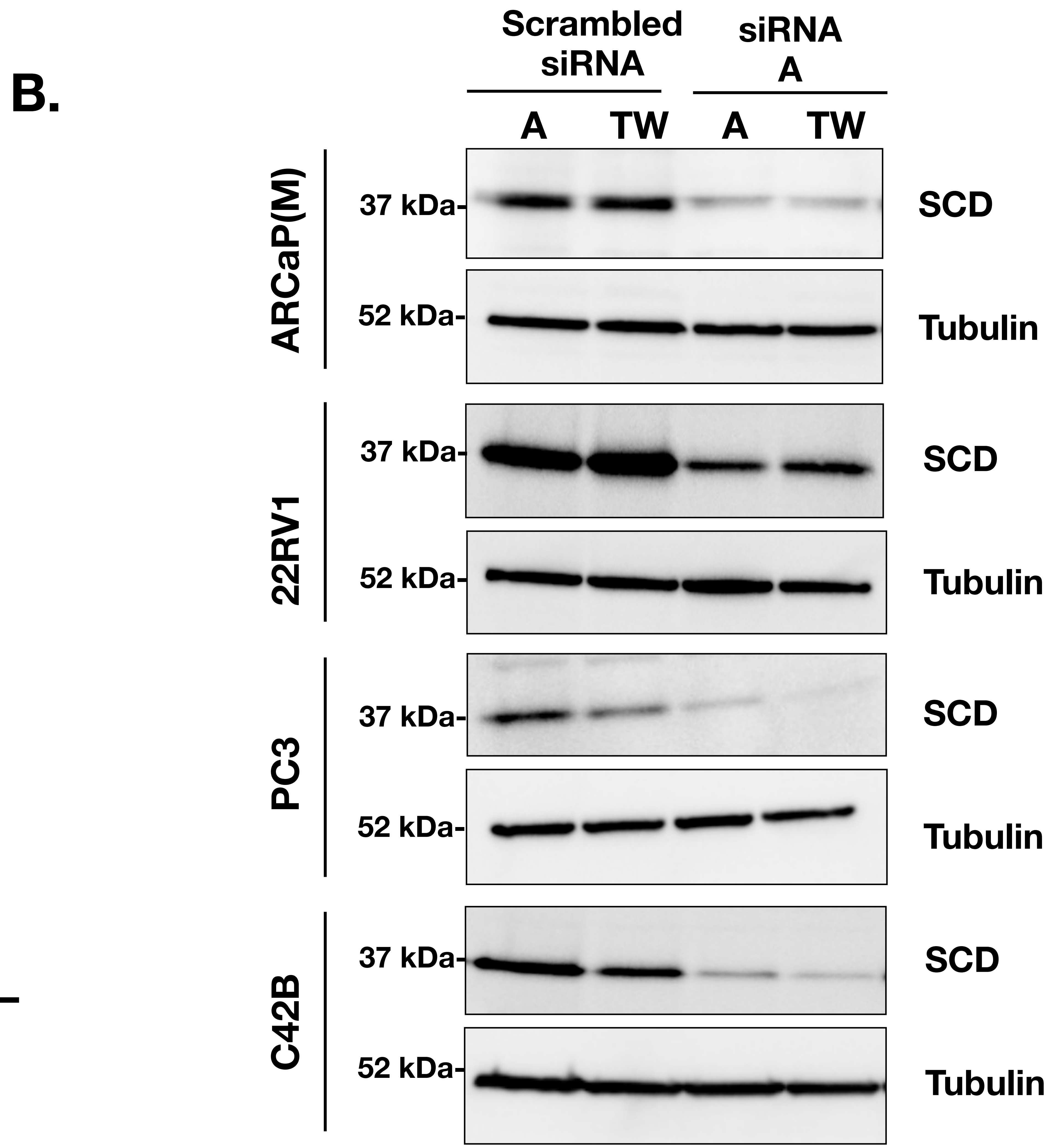

Supplementary Fig. 2

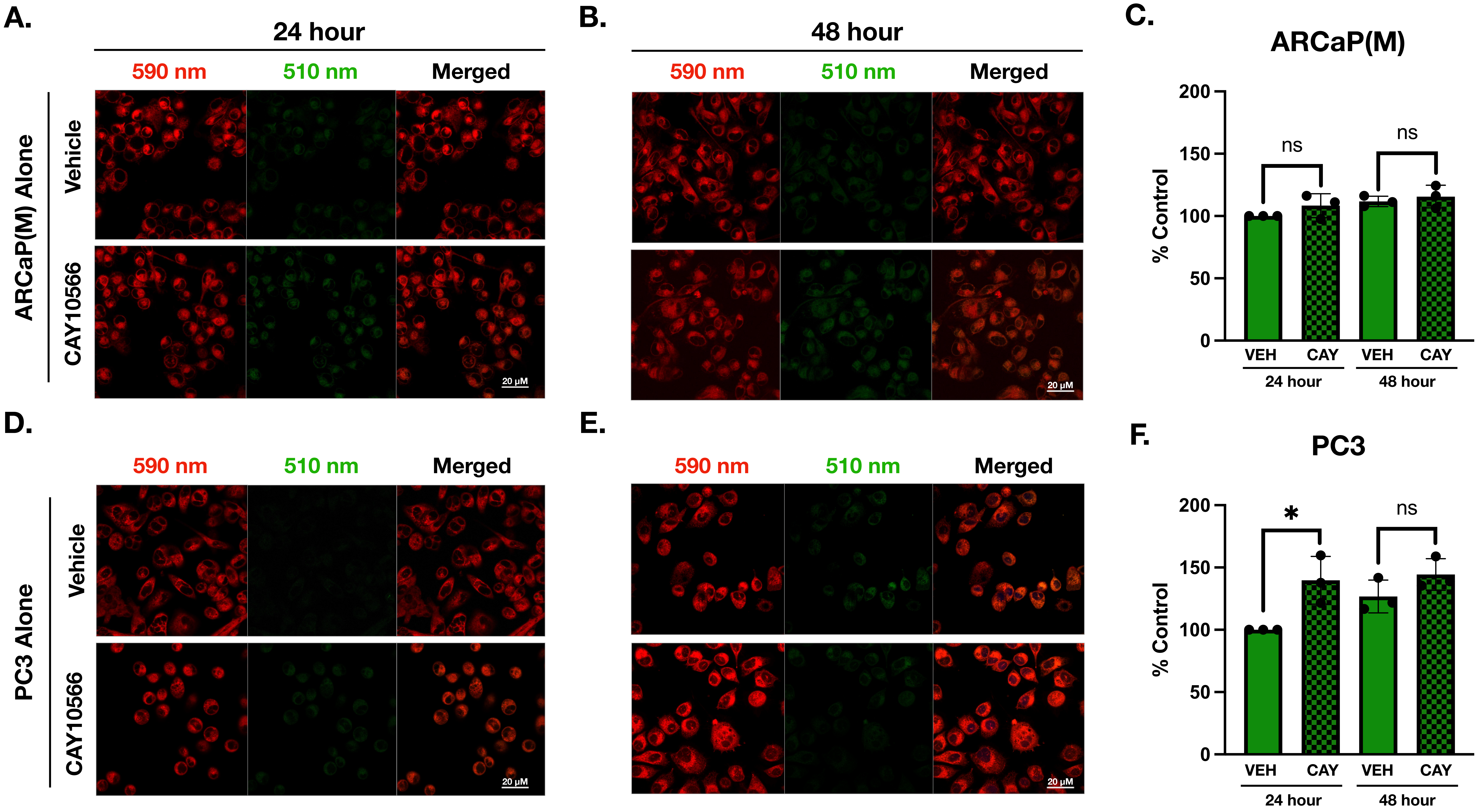

Supplementary Fig. 3

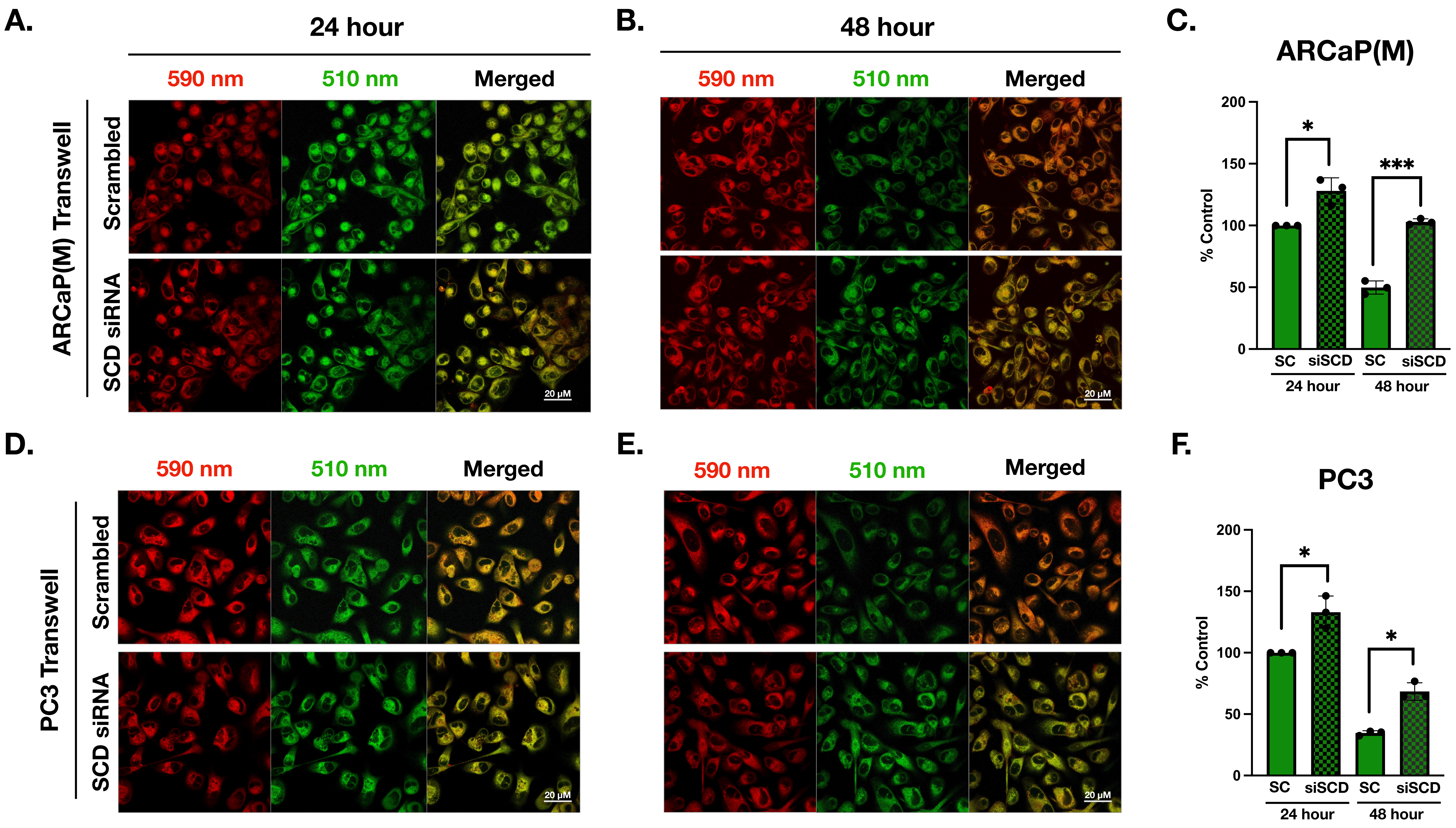

Supplementary Fig. 4

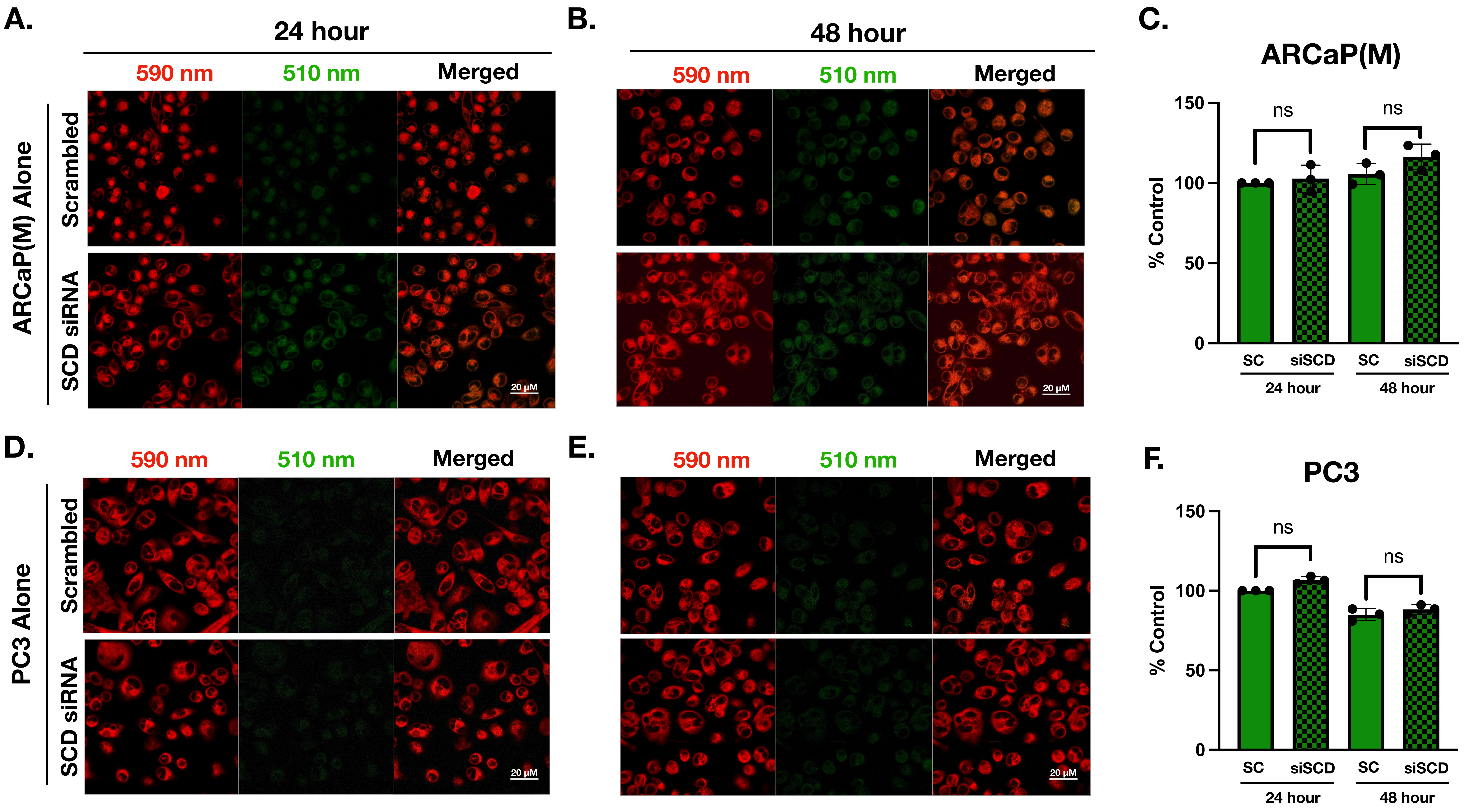

Supplementary Fig. 5

A.

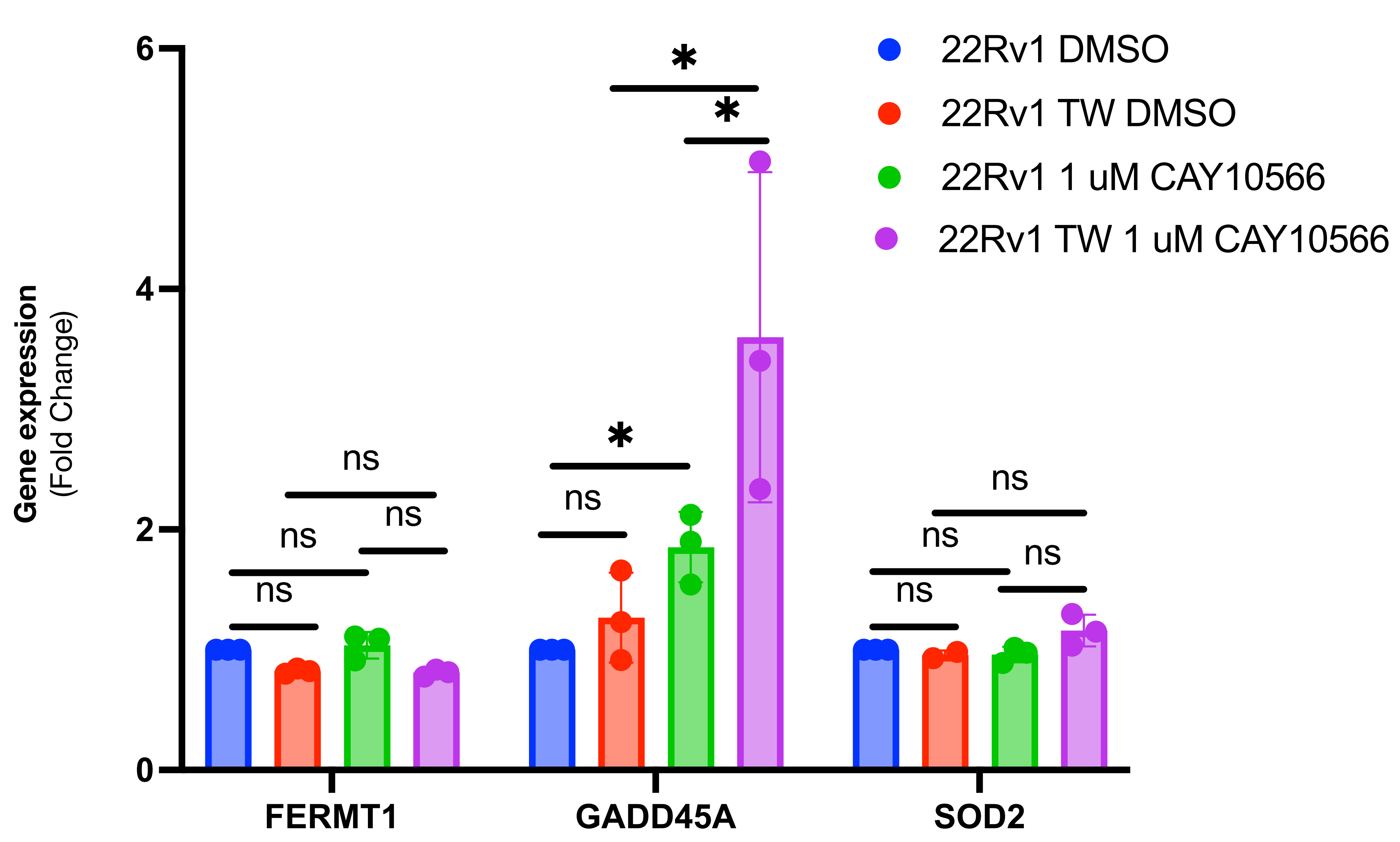

B.

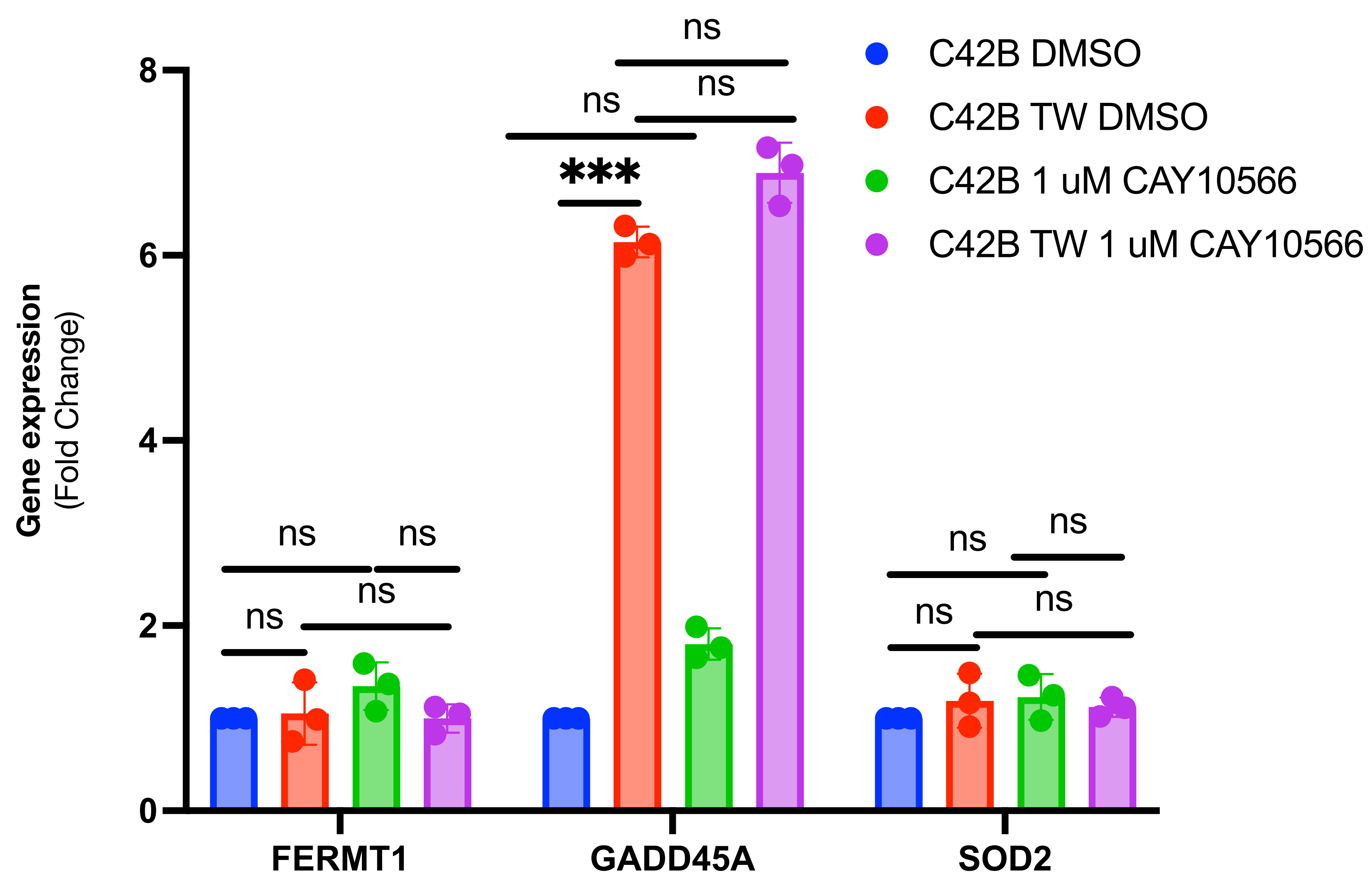

Supplementary Fig. 6

Supplementary Figure 7

ARCaP(M) Cells

ARCaP(M) Alone vs ARCaP(M) TW

A.

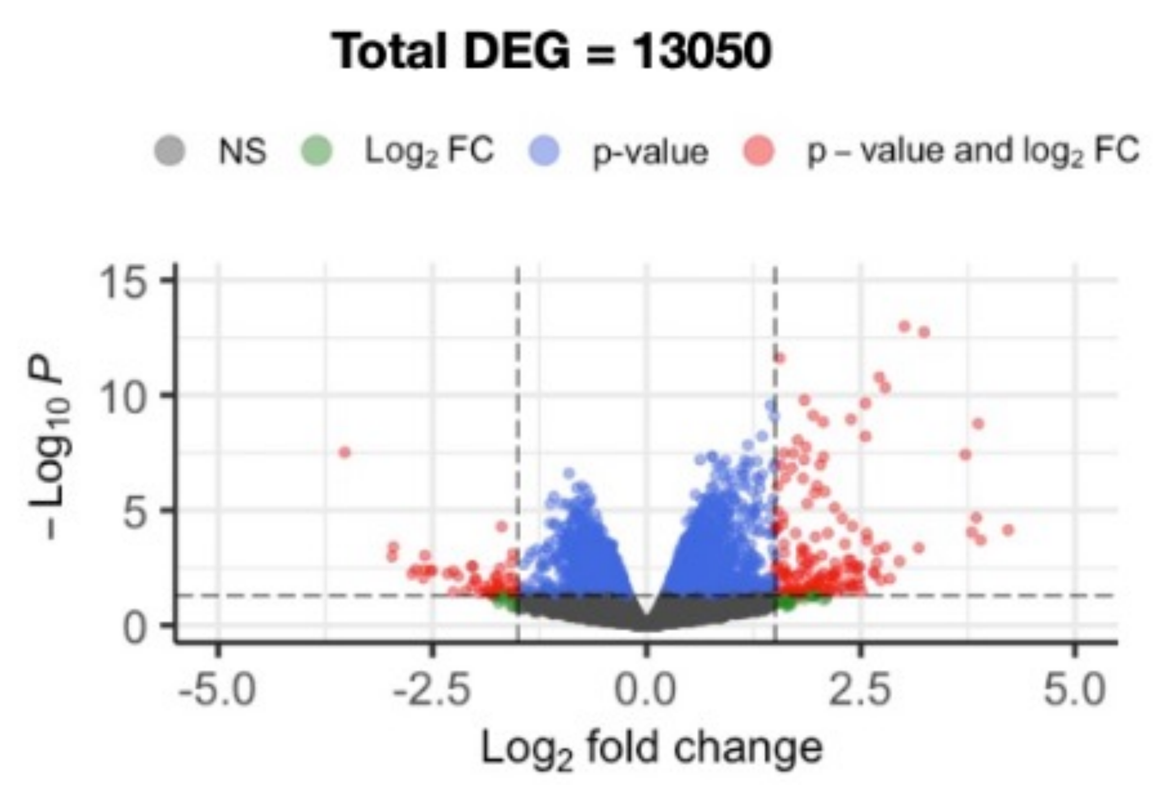

B.

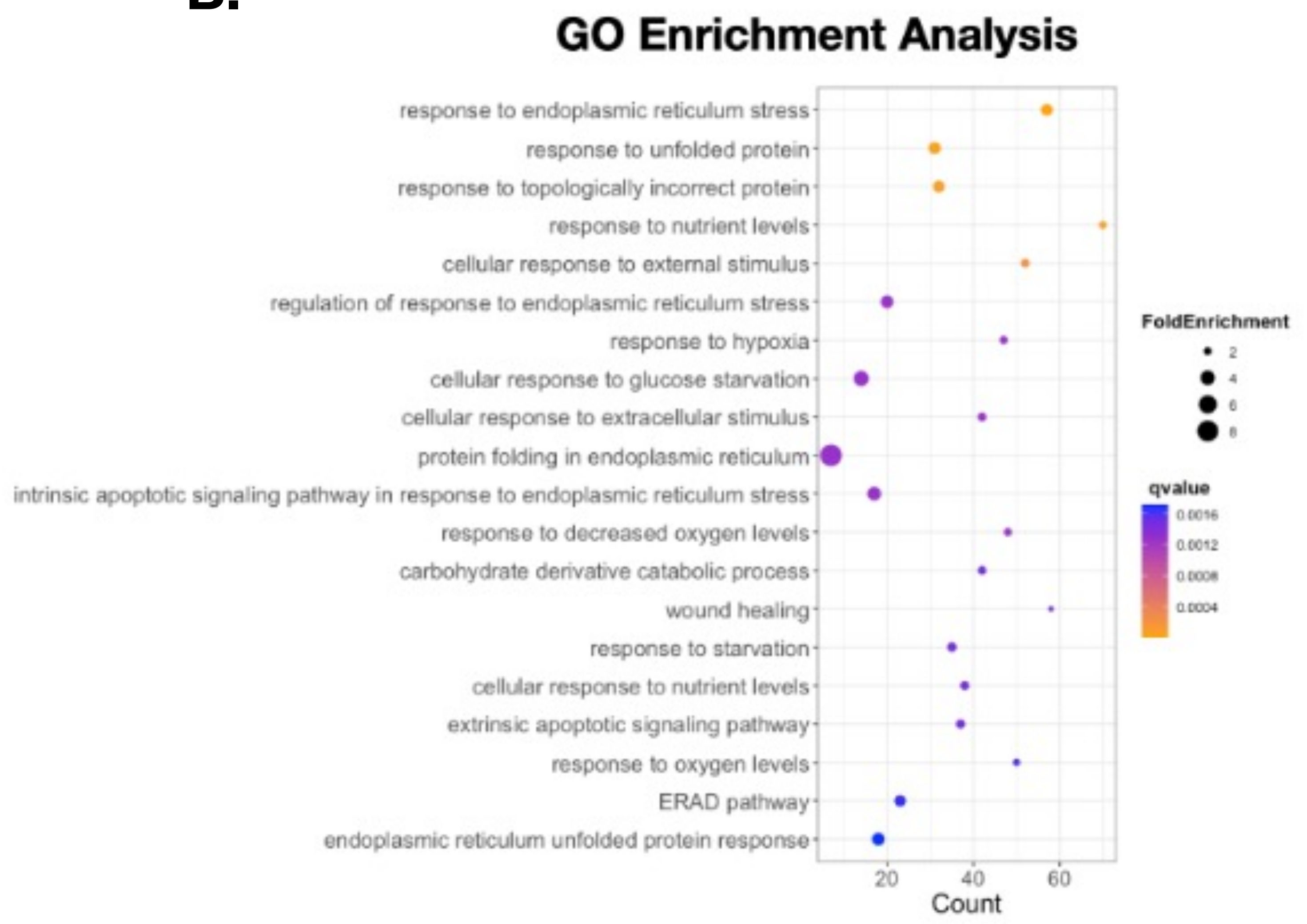

PC3 Cells

PC3 Alone vs PC3 TW

C.

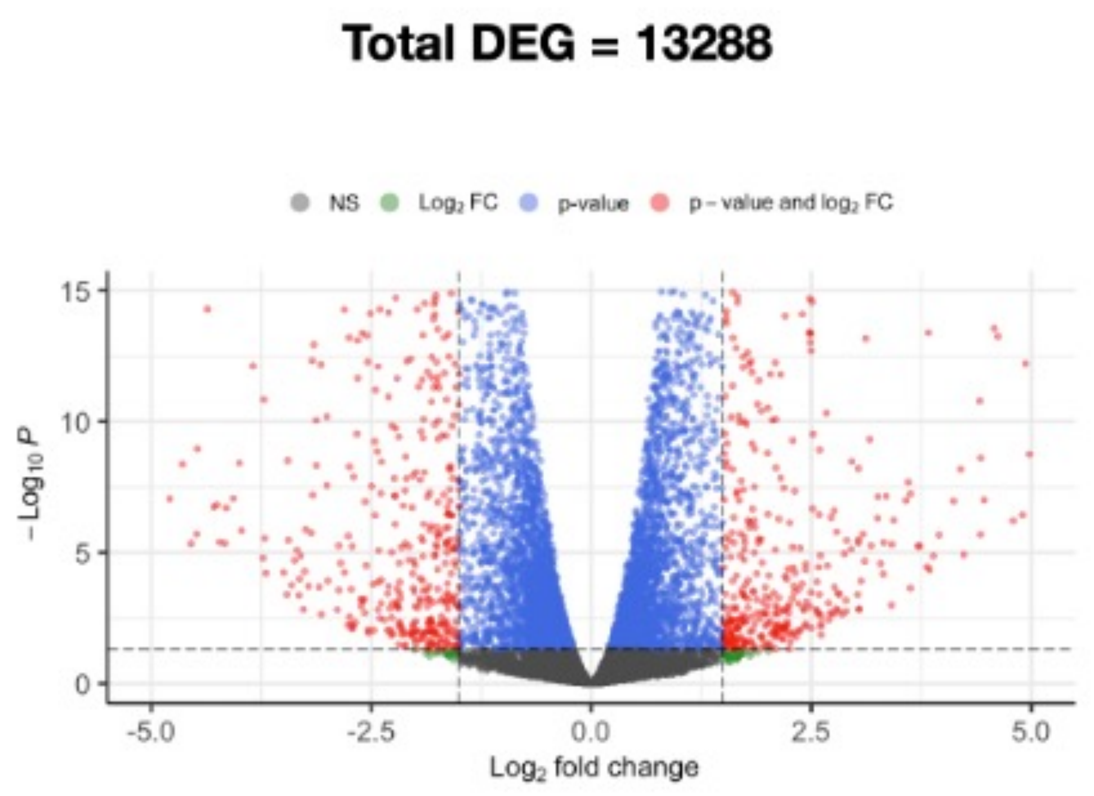

D.

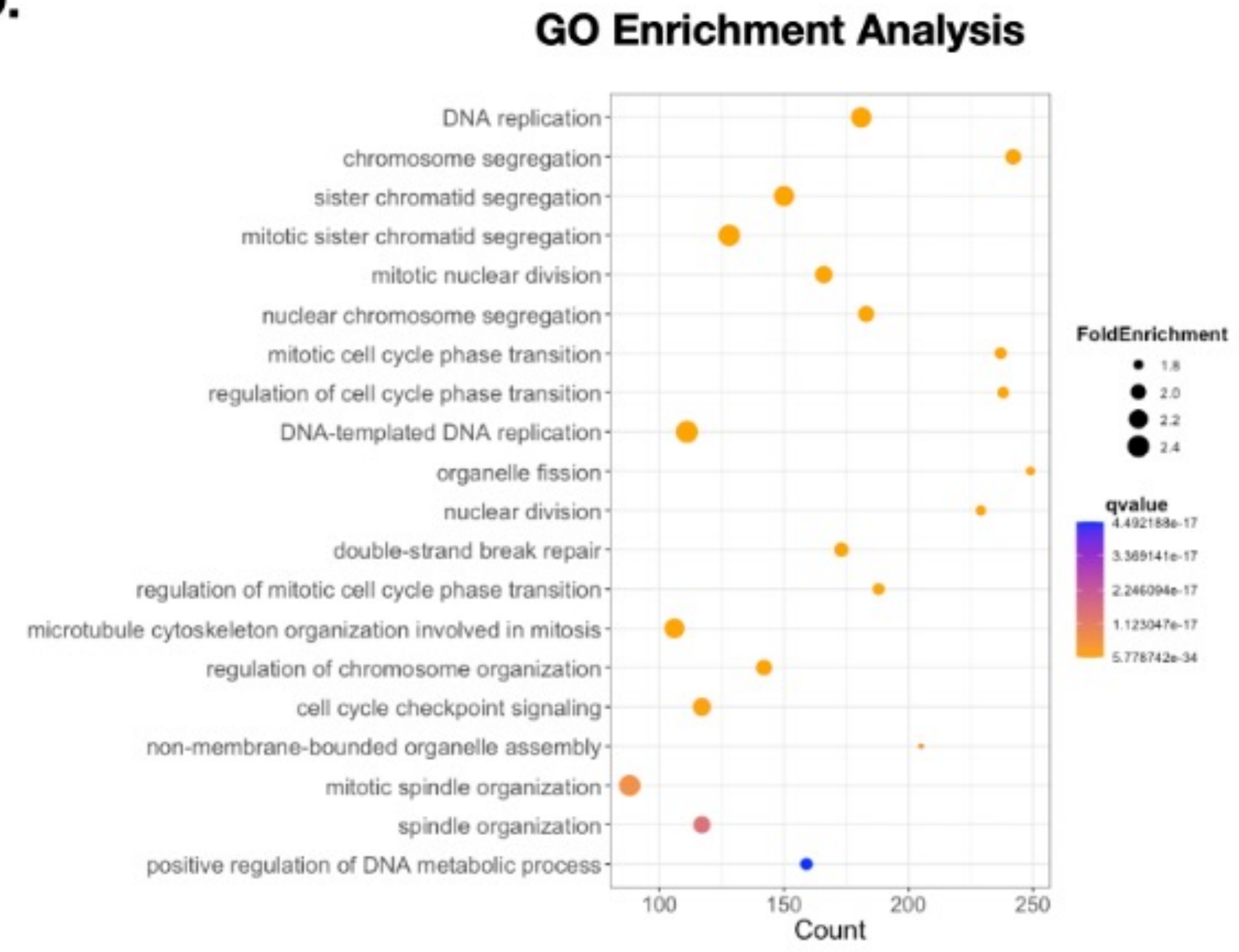

Supplementary Fig. 7

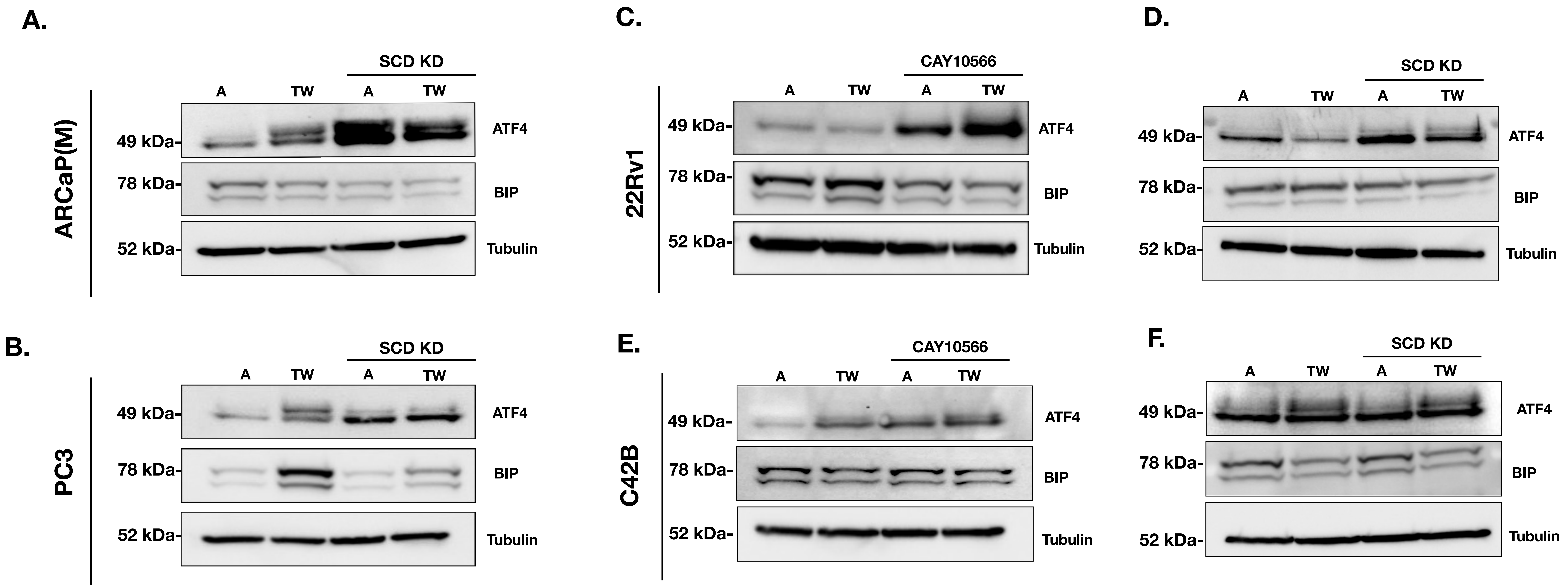

Supplementary Fig. 8

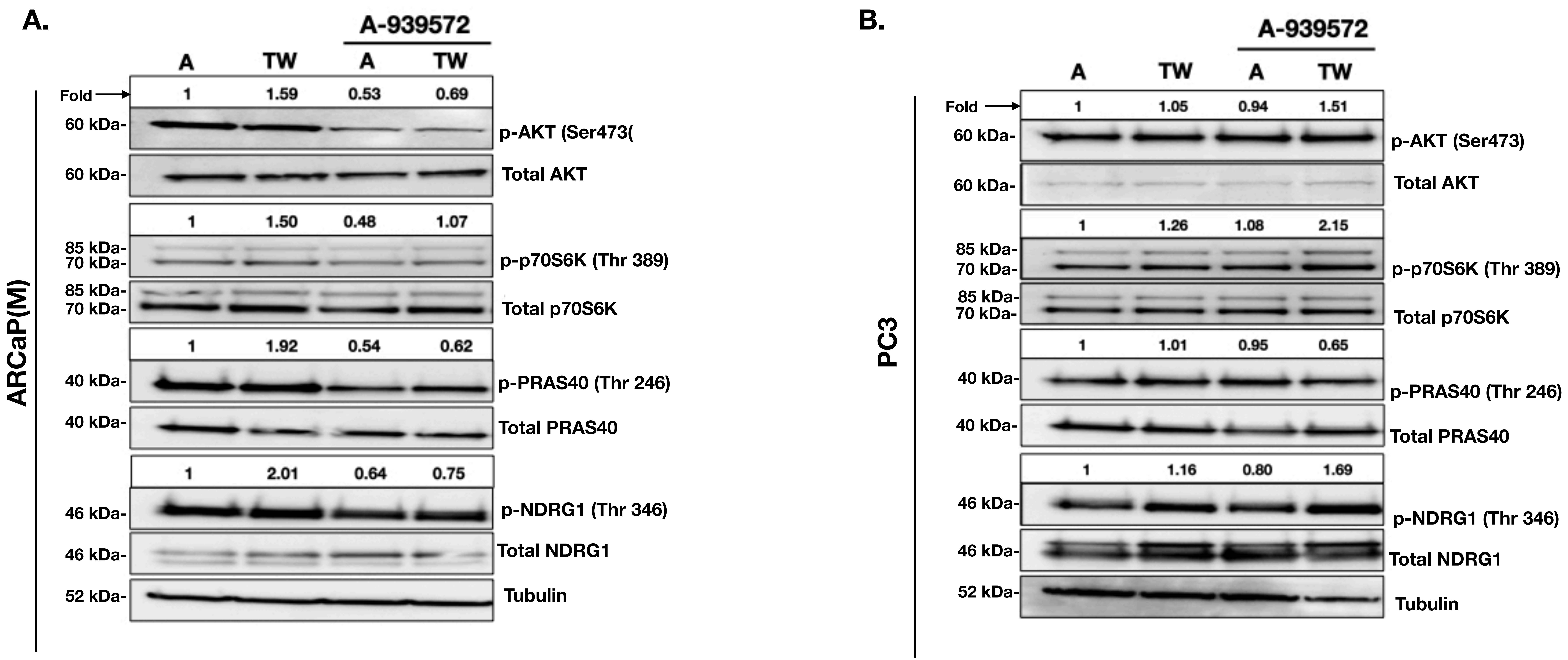

Supplementary Fig. 9

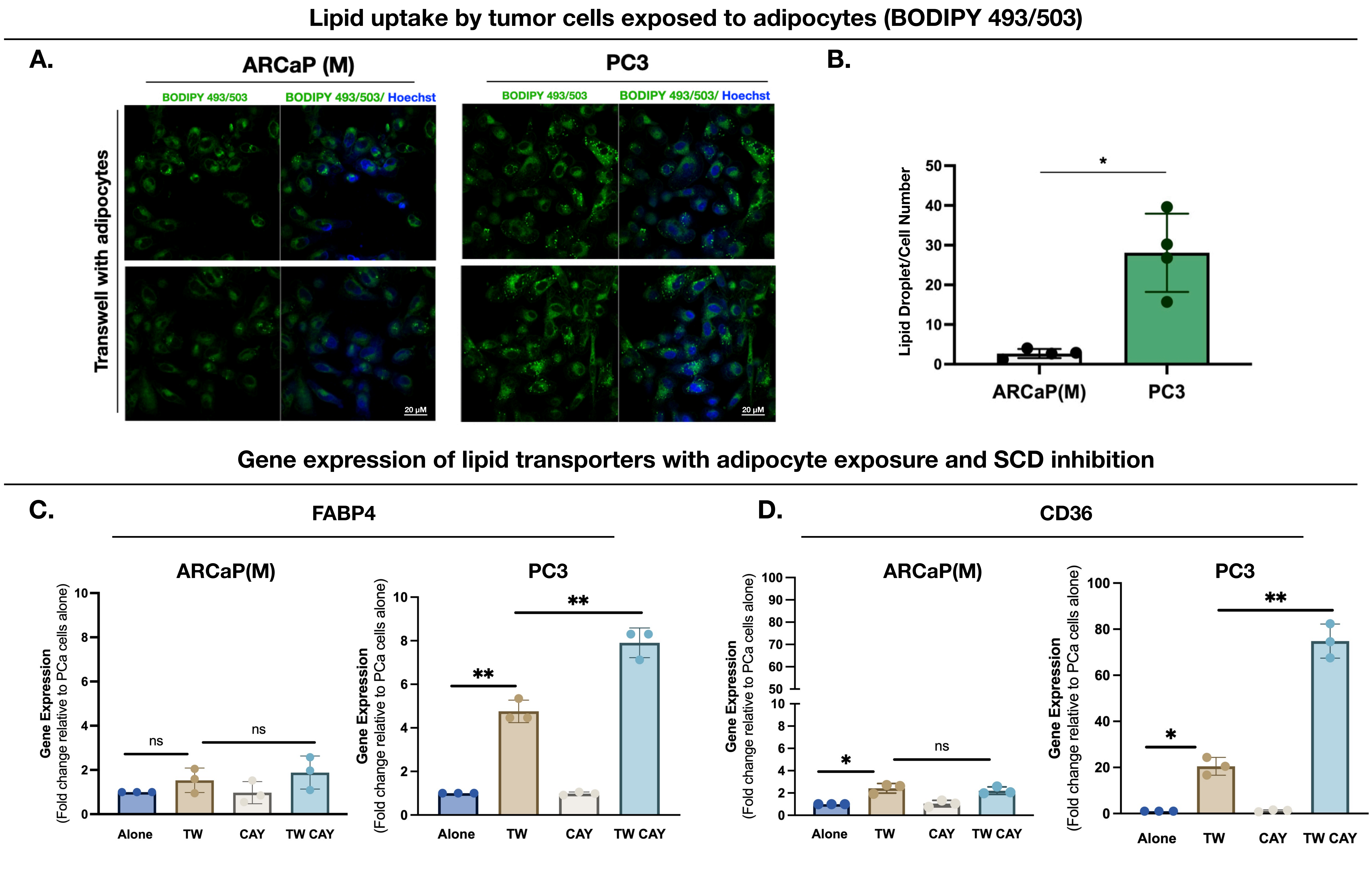

Supplementary Fig. 10

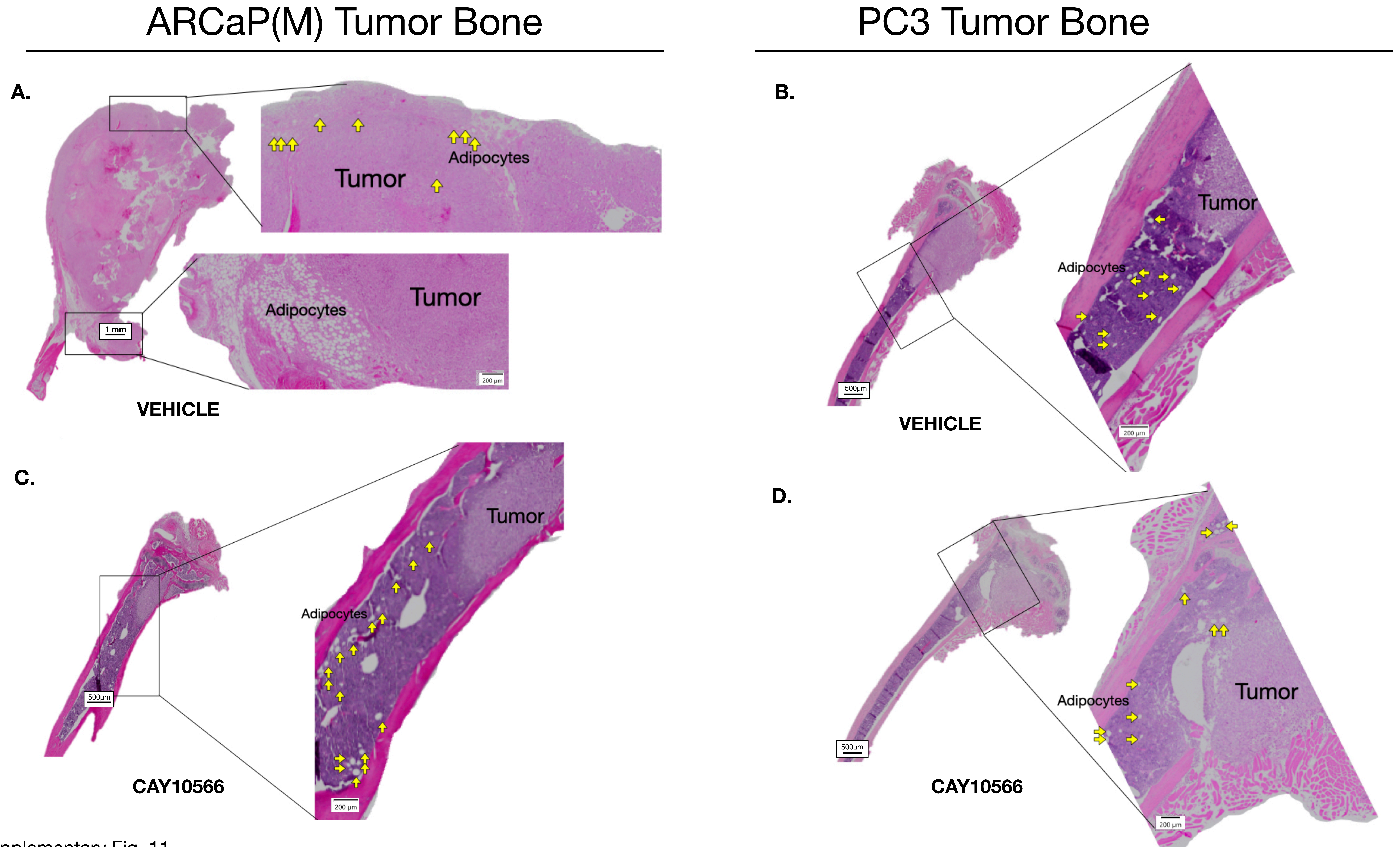

Supplementary Fig. 11

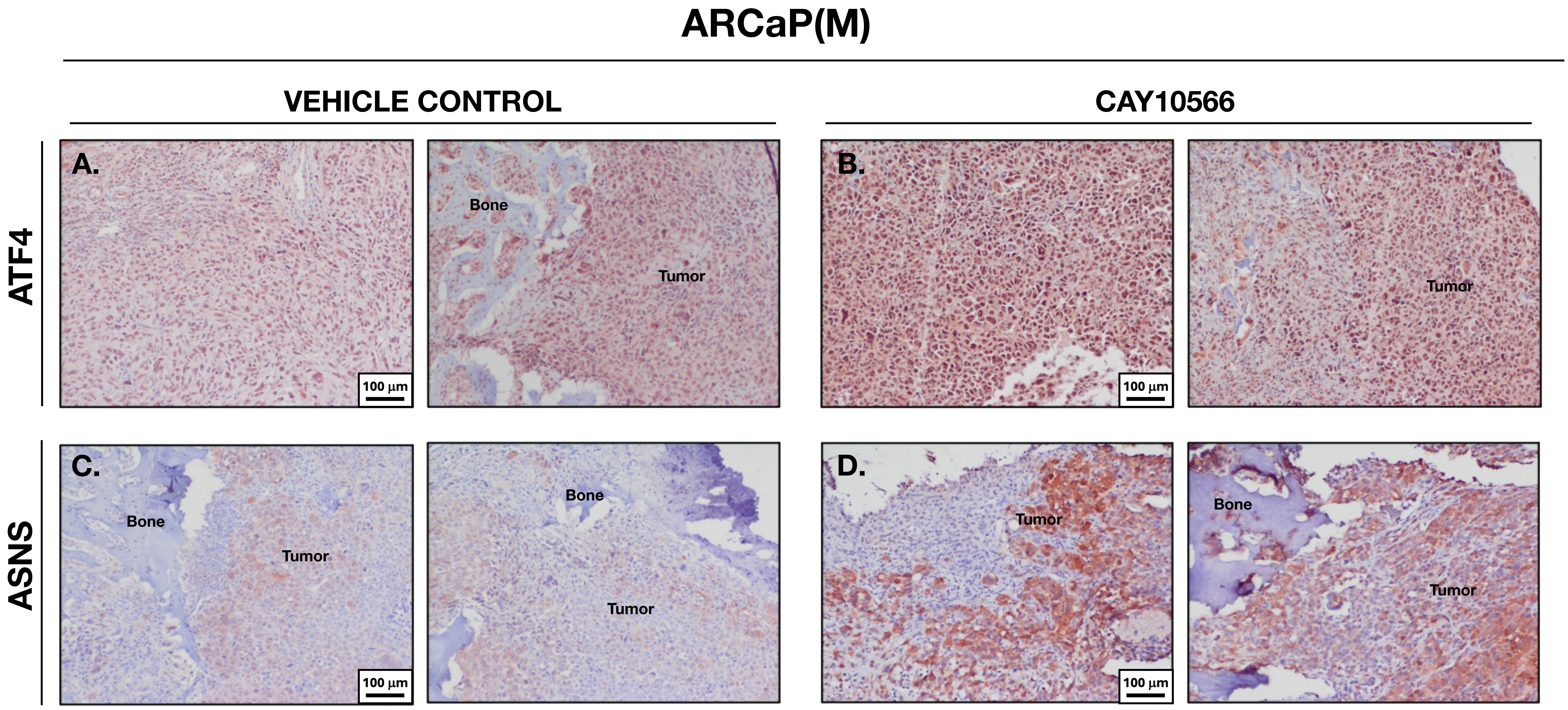

Supplementary Fig. 12

Supplemental Figure Legends:

Supplementary Figure S1. SCD is expressed in bone metastasis samples from PCa patients. A-I: Immunohistochemical (NovaRed) analysis of SCD (**A, C, E, G**) and Pan-Keratin (Type I) (**B, D, F, H**) expression in bone metastatic lesions from PCa patients. **A, B**: 4x images; **C-I**: 10x images; **I:** no primary antibody control. **J:** *SCD* mRNA levels in metastatic bone lesions from PCa patients; Relative levels were calculated using $2^{-\Delta Ct}$. Results for each patient are shown as a percentage of the housekeeping gene (*HPRT1*). Data are representative of 3 experiments. Scale bar: **A-B** = 200µm; **C-I** = 100µm.

Supplementary Figure S2. SCD knockdown by individual siRNA duplexes. A: *SCD* mRNA levels in ARCaP(M) cells upon treatment with nonoverlapping siRNAs targeting SCD as determined by TaqMan RT-PCR analysis. **B:** SCD protein levels in ARCaP(M), 22RV1, PC3 and C42B cells cultured alone (A) or in Transwell (TW) with adipocytes following treatment with scrambled siRNA or siRNA targeting SCD. The tubulin loading control for 22RV1 cells is identical to that shown in Figure 1 and Figure S8 because the same immunoblots were sequentially probed for multiple proteins. Data are representative of 3 experiments.

Supplementary Figure S3. SCD pharmacological inhibition does not increase lipid peroxidation in PCa cells grown in the absence of adipocytes. ARCaP(M) **(A-C)** and PC3 **(D-F)** cells were grown in alone conditions and treated with vehicle control (0.1% DMSO) or 1µM CAY10566 for 24 **(A, D)** and 48 **(B, E)** hours. BODIPY C11 staining was performed to examine lipid peroxidation (LPO) levels. An increase in LPO is indicated by a shift of fluorescence emission peak from ~590 nm (red) towards ~510 nm (green); 40x images. Quantification of ~510 nm shift for ARCaP(M) cells **(C)** and PC3 cells **(F)**. Data are representative of at least three experiments. Statistical analysis was performed using Student T-Test; * $p < 0.05$, ns = not significant. Error bars represent standard deviation (SD) of the mean.

Supplementary Figure S4. siRNA-mediated SCD knockdown increases lipid peroxidation levels in PCa cells in Transwell co-culture with adipocytes. ARCaP(M) **(A-C)** and PC3 **(D-F)** cells were treated with scrambled siRNA or siRNA targeting SCD and grown in Transwell (TW) co-culture with adipocytes for 24 **(A, D)** and 48 **(B, E)** hours. BODIPY C11 staining was performed to examine lipid peroxidation (LPO levels). An increase in LPO is indicated by a shift of fluorescence emission peak from ~590 nm (red) towards ~510 nm (green); 40x images. Quantification of ~510 nm shift for ARCaP(M) **(C)** and PC3 cells **(F),** indicating an increase in LPO upon treatment with siRNA targeting SCD as compared to scramble control. Data are representative of at least 3 experiments. Statistical analysis was performed using Student T-Test; *$p < 0.05$, *** $p < 0.001$. Error bars represent standard deviation (SD) of the mean.

Supplementary Figure S5. SCD pharmacological inhibition does not increase lipid peroxidation in PCa cells grown in the absence of adipocytes. ARCaP(M) **(A-C)** and PC3 **(D-F)** cells were grown in alone conditions and treated with vehicle control (0.1% DMSO) or 1µM CAY10566 for 24 **(A, D)** and 48 **(B, E)** hours. BODIPY C11 staining was performed to examine lipid peroxidation (LPO) levels. An increase in LPO is indicated by a shift of fluorescence emission peak from ~590 nm (red) towards ~510 nm (green); 40x images. Quantification of ~510 nm shift for ARCaP(M) cells **(C)** and PC3 cells **(F)**. Data are representative of at least 3 experiments. Statistical analysis was performed using Student T-Test; ns = not significant. Error bars represent standard deviation (SD) of the mean.

Supplementary Figure S6. SCD inhibition induces expression of GADD45A only in 22Rv1 cells. TaqMan RT-PCR analysis for mRNA expression of *FERM domain containing kindlin 1 (FERMT1), Growth Arrest and DNA Damage Inducible Alpha (GADD45A),* and *Superoxide Dismutase 2 (SOD2)* in 22Rv1 **(A)** and C42B **(B)** cells grown alone or in Transwell (TW) co-culture with adipocytes and treated with vehicle control (0.1% DMSO) or 1µM CAY10566. Data represent 3 experiments Statistical analysis was performed using Student T-Test; *$p < 0.05$, *** $p < 0.001$, and ns = not significant. Error bars represent standard deviation (SD) of the mean.

Supplementary Figure S7. Differential gene expression and GO Enrichment Analysis of PCa cells in monoculture compared to in Transwell co-culture with marrow adipocytes. A: EnhancedVolcano plot indicating the differentially expressed genes (DEG) in ARCaP(M) cells grown in alone conditions or in the presence of bone marrow adipocytes (TW), and **B:** Gene Ontology (GO) Biological Process enrichment analysis of significant DEGs ($p < 0.02$) identified in ARCaP(M) cells using enrichGO. **C:** EnhancedVolcano plot indicating the differentially expressed genes (DEG) in PC3 cells grown in alone conditions or in the presence of bone marrow adipocytes (TW), and **D:** Gene Ontology (GO) Biological Process enrichment analysis of significant DEGs ($p < 0.02$) identified in PC3 cells using enrichGO. The count on the x-axis depicts the number of genes in each pathway; Fold Enrichment determines the dot size, and the gradient color indicates the q-value; darker color indicates a lower q-value.

Supplementary Figure S8. SCD knockdown/inhibition increases ER stress levels in PCa cells. Immunoblot analysis of ER stress markers Activating Transcription Factor 4 (ATF4) (top) and Binding Immunoglobulin Protein (BIP) (middle) in ARCaP(M) **(A)**, PC3 **(B)**, 22Rv1 **(C, D)** and C42B **(E, F)** cells grown alone (A) or in Transwell (TW) co-culture with adipocytes and treated with vehicle control (0.1 % DMSO or scrambled siRNA), 1µM CAY10566 **(C, E)** or siRNA targeting SCD **(A, B, D, F)**. The tubulin loading control for 22RV1 cells in panel D is identical to that shown in Figure S2B because the same immunoblots were sequentially probed for multiple proteins. Data are representative of at least 3 experiments.

Supplementary Figure S9. Inhibition of SCD decreases mTOR signaling in ARCaP(M) but not PC3 cells. ARCaP(M) **(A)** and PC3 **(B)** cells were cells grown alone (A) or in Transwell (TW) co-culture with adipocytes and treated with vehicle control (0.1% DMSO) or 250μM A-939572 for 48 hours and subjected to immunoblot analysis of total and phosphorylated downstream mTOR proteins: AKT Serine/Threonine Kinase 1 (AKT), p70 ribosomal protein S6k kinase (P70S6K), Proline-Rich AKT Substrate 40 kDa (PRAS40), and N-myc downstream regulated gene (NDRG1). Data are representative of represent at least3 experiments.

Supplementary Figure S10. PC3 cells exposed to adipocytes have increased lipid uptake and expression of lipid transporters, which are further increased by SCD inhibition. A: Immunofluorescence imaging of lipid droplets (BODIPY 493/503; green fluorescence) in ARCaP(M) and PC3 cells grown in Transwell (TW) co-culture with adipocytes for 48 hours; 40× images; Hoechst dye (blue) was used to stain the nuclei. **B:** Quantification of lipid droplet number per cell was determined using ImageJ's analyze particles function. **C, D:** TaqMan RT-PCR analysis of *Fatty Acid Binding Protein 4 (FABP4)* **(C)** and *Cluster of differentiation 26 (CD36)* **(D)** in ARCaP(M) and PC3 cells grown alone or in TW co-culture with adipocytes in the absence or presence of 1μM CAY10566 (CAY). Data are representative of at least 3 experiments. Statistical analysis was performed using Student T-Test; *$p < 0.05$, ** $p < 0.01$, and ns = not significant. Error bars represent standard deviation (SD) of the mean. Scale bar = 20μm.

Supplementary Figure S11. Loss of adipocytes during tumor progression in bone. Hematoxylin and eosin (H&E) staining showing adipocyte content in the bone marrow of ARCaP(M)- and PC3-tumor–bearing mice treated with vehicle (**A, B**) or the SCD inhibitor CAY10566 (**C, D**). Marrow adiposity was induced by high-fat diet (HFD) prior to tumor implantation. Remaining adipocytes within the marrow or tumor space are indicated by yellow arrows. A large cluster of adipocytes observed in a vehicle-treated ARCaP(M) tumor that breached the bone is located near the distal tibia and likely represents non-regulated (constitutive) adipocytes rather than marrow adipocytes responsive to lipolytic stimuli. Data are representative of 3 experiments. Scale bar: Full images; **A** = 1mm, **B-D** = 500μm; Zoomed in images; **A-D** = 200μm.

Supplementary Figure S12. SCD inhibition *in vivo* increases ER stress and expression in ARCaP(M) bone tumors. High-fat diet (HFD)-fed mice were intratibially implanted with ARCaP(M) cells and treated with vehicle control (5% DMSO in corn oil) or CAY10566 (5mg/kg) by oral gavage after confirmation of tumor formation. **A-D:** Immunohistochemical (NovaRed) staining of Activating Transcription Factor 4 (ATF4) **(A, B)** or Asparagine Synthetase (ASNS) **(C, D)** protein in bone tumors from vehicle-treated (**A, C**) and CAY10556-treated (**B, D**) mice; 10× images. Tumor, bone, and marrow are noted in images. Data representative of 3 experiments. Scale bar = 100μm.
